# Active commute to school: does distance from school or walkability of the home neighbourhood matter? A national cross-sectional study of children aged 10–11 years, Scotland, UK

**DOI:** 10.1136/bmjopen-2019-033628

**Published:** 2019-12-23

**Authors:** Laura Macdonald, Paul McCrorie, Natalie Nicholls, Jonathan R Olsen

**Affiliations:** MRC/CSO Social and Public Health Sciences Unit, University of Glasgow, Glasgow, UK

**Keywords:** walkability, active travel to school, children, dwelling density, intersection density, GIS

## Abstract

**Objectives:**

To study the extent to which home-to-school distance and neighbourhood walkability were associated with self-reported active travel to school (ATS) (eg, walking, cycling), and to explore how distance moderates the effect of walkability on ATS, among 10–11 years old.

**Design:**

Cross-sectional study.

**Setting:**

Data were collected between May 2015 and May 2016 in partnership with the Growing Up in Scotland Study, a nationally representative longitudinal cohort study.

**Participants:**

713 children (male (n=330) and female (n=383) 10–11 years old) from Studying Physical Activity in Children’s Environments across Scotland.

**Primary and secondary outcome measures:**

Children who actively travelled to/from school categorised as active all (100% of ATS) and active 60%+ (at least 60% of ATS); home-to-school road/path network distance (<0.5 km, 0.5 to <1 km, 1 to <1.5 km, 1.5 to <2 km, 2 km+); home neighbourhood walkability (i.e., composite measure of road/path intersection density and dwelling density) (in quintiles).

**Results:**

Distance and walkability were both associated with ATS. The likelihood of ATS for all or most journeys decreased with increasing distance. Compared with ‘most’ walkable areas (Q1), the odds of active all were significantly lower within least walkable areas (Q5 OR 0.45, 95% CI 0.21 to 0.99), and odds of active 60%*+* were significantly less in Q2–Q5 (lowest odds Q5 OR 0.20, 95% CI 0.07 to 0.47). Regarding walkability and distance interactions, for all distance categories, higher walkability increased the probability of ATS (for most school journeys).

**Conclusion:**

Walkability was positively associated with ATS within all distance categories, with the relationship between walkability and ATS more complex than the clear-cut association between distance and ATS. A more walkable environment had a larger effect on the probability of reaching the 60% threshold of school journeys using ATS than the probability of always travelling in an active manner. Investment is needed in existing less walkable neighbourhoods to provide infrastructure to support opportunities for children’s ATS.

**Strengths and limitations of this study**

- This study used a sample of children from across the whole of Scotland, and was weighted to ensure representativeness to the wider population of 10–11 years old living within Scotland.
- We included objectively measured home-to-school distance and walkability score.
- We explored how home-to-school distance moderates the effect of walkability on active travel.
- Travel mode (active vs non-active) was determined via self-report rather than objective measurement.

## Introduction

Physical inactivity among children is a considerable public health concern. Globally, health authorities recommend that physical activity for children aged 5–17 should reach a minimum of 60 min of moderate-intensity to vigorous-intensity daily.^1^ High levels of inactivity among children are seen in the UK,^2^ Europe^3^ and the USA.^4^ It is estimated that around two-thirds of Scottish children do not meet recommended activity levels, with levels falling with increasing age.^5^ Greater overall activity levels have been observed among children who actively travel to and from school.^6–8^ Active travel to school (ATS) can provide children with health, academic and psychological benefits,^9^ and can promote social interaction and cognition, such as acquiring spatial knowledge.^10^ However, only around 50% of children in Scotland walk, scoot or cycle to school, and this proportion has slightly decreased in the last 10 years.^11^ This has been noted elsewhere in the UK and Scandinavia,^12^ Australia,^13^ the USA^14^ and Canada.^15^

The existing literature seeks to explore the factors associated with children and parents selecting non-active versus active modes for travel to school. Various studies have modelled ‘walkable’ neighbourhood design or ‘walkability’ and examined how built environment features of the neighbourhoods around schools influence ATS, however, few UK-based studies exist.^16^ Most studies conducted to date tend to be based in the USA,^17–21^ Canada,^22–26^ Australia,^27–30^ New Zealand^31–35^ and Spain.^36–38^

A number of factors are examined in the existing literature, such as ‘home-to-school distance’ (shorter distance, more convenient for ATS), ‘residential density’ (higher densities; areas less car dependent and more convenient for walking) and ‘street connectivity/intersection density’ (higher densities; route more direct and quicker); with density measures often included within customised ‘walkability scores’.

Various papers have demonstrated home-to-school distance to be an essential factor for ATS, with those living closer to their school more likely to use active means.^20 24 27 32 34 36 37 39–45^ Findings for the influence of residential density on ATS vary; it was seen to positively influence ATS when included within a composite walkability score in a number of studies.^22 27 37 38 42 46^ However, other studies found no such relationship^18 34 35^ or detected a negative association between residential density and ATS.^23 24^ The results also varied for associations between intersection density and ATS: a number of papers found that the rates of ATS were higher within neighbourhoods with greater intersection densities,^25 27 34 37 38 42 44^ while other research did not find an association^23 32 35^ or uncovered a negative relationship.^24^ Southern Denmark research combined measures of road connectivity, residential density and traffic exposure within a walkability index, and found that the index was positively associated with ATS, and that distance-to-school moderated the association.^42^ Walkability and distance interactions have been little studied in the existing ATS research; inclusion of these interactions allows exploration of whether the built environment influences travel mode among those who live nearer, and those who live further, from school.

Our previous work showed that walkability features—road/path intersection density and dwelling density, combined into a composite score—varied by small area deprivation.^47^ We found that catchment areas around primary schools in more deprived areas of Scotland were more walkable, with walkability decreasing as areas became more affluent. However, this work did not explore associations between the walkability score and ATS rates. The spatial variation in built environment features warrants the study of links to potential variation in ATS rates. We thus, took the opportunity within the current study to use existing data on children’s ATS from the Studying Physical Activity in Children’s Environments across Scotland study^48^ and investigate the relationship with the composite densities score (ie, road/path intersection density and dwelling density). Furthermore, within this current paper, we include additional factors potentially related to mode of travel to school, such as home-to-school distance, and season and latitude (as proxies for weather conditions). Additionally, we go beyond the majority of existing studies by exploring interactions between walkability and distance-to-school. To our knowledge, this is the first study of its kind based in the context of Scotland.

Our aim is, thus, to examine associations between child self-reported ATS and objectively measured home-to-school distance and neighbourhood walkability score in the context of Scotland, with and without adjusting for confounders (urban/rural, household income and weather conditions). Additionally, we aim to explore how the effect of walkability density score on active transport levels is moderated by home-to-school distance.

## Methods

### Participants

We analysed data from participants in the SPACES study.^48^ The aim of SPACES was to explore the environmental determinants of physical activity by conducting a large-scale, nationally representative, accelerometer and global positioning systems (GPS) observational study.^49^ The participants involved in SPACES were recruited from the Growing Up in Scotland (GUS) study, a nationally representative longitudinal cohort study originating in 2005. As part of sweep 8 interviews (conducted September 2014 to February 2015), parents and children were provided with brief information about SPACES and asked if their contact details could be passed on to SPACES staff. From a possible 2402 children, who had participated in GUS sweep 8 interviews, 90% (n=2162) of parents consented to be contacted, and study information, registration documents and consent forms were sent by post using the main carer as primary contact. The data collection for SPACES took place between May 2015 and May 2016. The children were asked to complete a travel diary to gather data on how they travel to and from school each day during two school weeks (10 days/20 trips). The children completed the travel diaries at home under the supervision of their parent/carer. The child’s home and school location were collected.

### Active travel to school

Within the travel diary, children recorded the amount of time (in minutes) they spent travelling by transportation mode from the following nine options: walking, cycling, car, bus, train, scooter, skateboard, ferry and tram. We created an ATS variable for any school journeys that included a stage recording travel by foot, cycling, scooter and/or skateboard. Where a single school journey included a combination of travel modes the main form of travel was chosen, for example, a 1 min walk to a bus stop and a 10 min bus journey would be categorised as a non-active school journey overall. A total of 713 children provided travel diary data for up to 20 trips (10 trips to school and 10 trips home from school over a 2-week period), and an additional 22 children who did not provide any travel diary data were excluded from analyses. The minimum number of school journeys recorded in the travel diary was two (as recorded by three children who travelled 100% of school journeys actively); 90% of children recorded travel for ten or more school journeys.

Further categories of using an active mode were created: active all if all school journeys recorded were active; and active 60%*+* if 60% or more of school journeys were active. The cut-off of 60% was chosen based on its previous use to represent usual or habitual mode of travel.^29^

### Walkability

Walkability scores were calculated for the whole of Scotland at data zone level for 2015 as a product of intersection density (connectivity) and dwelling density (both have previously been linked to ATS, see Macdonald *et al* for information). Street network and path network datasets for Scotland in 2015 were obtained from EDINA Digimap.^50^ A count of the number of dwellings and the land area in hectares for each data zone (for 2015) were acquired from the Scottish Government.^51^ ArcMap V.10.3 was used to calculate intersection density using the ratio of the number of true intersections (three or more legs) to the data zone area.^52^ Z-scores were computed using IBM SPSS Statistics V.21 for both variables to standardise scores, and the following formula used: Walkability score= (2×intersection z-scores)+(dwelling density z-scores) (a similar formula to that used by Frank *et al* 52). Connectivity was weighted more heavily as previous work highlights the strong influence of this measure on active travel choices.^53^ Data zone walkability scores were divided into quintiles (Q) (Q1: most walkable to Q5: least walkable). Each child’s home location was linked to the walkability score quintile for the data zone in which the home was located.

### Distance from home to school

Exact spatial coordinates of home and school addresses were obtained using an online batch geocoder.^54^ The network distance (km) was calculated from each child’s home location to their school using the gmapsdistance package^55^ within R V.3.2.0 in February 2018. The software calculated the shortest distance between these two precise geolocations using the Google Maps road and path network, for a walked school journey. Distances were grouped into the following five categories: <0.5 km, 0.5 to <1 km, 1 to <1.5 km, 1.5 to <2 km and 2 km or more.

### Statistical analysis

For our analysis, we reported two key outcomes of reported travel to and from school:

1. All school journeys recorded as active (active all).
2. Sixty per cent or more of school journeys included at least one active mode (active 60%*+*).

Participant characteristics were recorded in questionnaires, as well as season of measurement (ie, data collected in spring, summer, autumn or winter) and number of school journeys. Car ownership was not included in analysis as almost all households had access to at least one car (98%). Each key outcome was described by distance to school, walkability and income. Cross-tabulations with Pearson’s X^2^ were used to test for any statistical differences between categories within these groups and ATS.

We employed an analysis plan using a three-stage model to investigate the association between active travel and two built environment characteristics, home-to-school distance and walkability, separately (models 1 and 2), and then combined (model 3). This type of three-stage model is commonly used when describing the investigation of neighbourhood characteristics separately and combined.^56^

The models were thus performed separately for:

1. Home-to-school distance (unadjusted and adjusted).
2. Walkability score (unadjusted and adjusted).
3. Walkability score and home-to-school distance combined (adjusted).

Logistic regression models were performed and reported the probability of active all and active 60%+ by home-to-school distance and/or walkability score of home area. The models were performed unadjusted and adjusted for urban/rural (home) classification, household income, season of measurement and latitude. The sixfold urban/rural classification (2014) (based on the population size and accessibility) by postal code was downloaded from the Scottish Government website,^57^ household income data were collected from the SPACES parental/carer questionnaire, season of measurement obtained from travel diaries and latitude extracted from home address. Urban/rural and income were included as confounders as ATS rates differ by these variables; children from lower-income households, and children within urban areas, are more likely to use active means to travel to school.^58^

We included season and latitude as potential confounders as they are linked to weather conditions which may influence mode of travel to school; rain and/or low temperatures have been cited as barriers to active travel in previous research.^25 36 59^ Sunshine duration and average temperature decrease with increasing latitude, while average rainfall varies by season, that is, rainfall is the highest in the autumn and the winter and lower in the summer and the spring.^60^ Previous qualitative research found that Scottish school children aged 10–13 years old, frequently cited poor weather, such as cold temperature or rain, as a barrier to ATS.^59^

To explore how the effect of walkability score on active transport levels might be moderated by home-to-school distance, the interaction between the two was included in model three. From these models, interaction plots were produced, illustrating the predicted probability of active all and active 60%*+* by each distance category, at different values along the range of walkability scores.

Analyses were conducted using STATA V.14.2 (STATA), and accounted for the clustered and stratified survey sample design of the GUS cohort. Sampling weights were applied to allow for non-consent to contact, and non-consent and non-compliance of those invited to take part. Detailed information on the clustered and stratified survey sample design, and sample weighting survey, are available within online supplementary text (and further information is available here, Bradshaw *et al* 61).

10.1136/bmjopen-2019-033628.supp1 Supplementary data

Strengthening the Reporting of Observational studies in Epidemiology cross-sectional reporting guidelines were adhered to within this study.^62^

### Patient and public involvement

We did not involve study participants in the development of the research question, design and implementation of the study or interpretation of the results.

## Results

A total of 713 children completed travel diaries and were included in the analysis. Around 60% lived in urban areas, 53.7% were girls and 50% (of diaries) were completed in autumn (table 1).

**Table 1:** Participant summary descriptive statistics (unweighted and weighted) (n=713)

Summary descriptive statistics	Unweighted	Weighted
	% (of group)	% (of group)
Sex		
Male	46.3	46.0
Female	53.7	54.0
Age		
10	33.0	34.5
11	67.0	65.5
Urban rural classification (sixfold)		
Large urban areas	29.5	33.8
Other urban areas	29.9	34.1
Accessible small towns	10.8	8.8
Remote small towns	3.2	2.5
Accessible rural	17.5	13.7
Remote rural	9.1	7.2
Total household income		
<£19 999 pa	10.1	21.9
£20.000–£28.999 pa	10.8	17.9
£29.000–£37.999 pa	15.7	14.8
£38.000–£49.999 pa	17.5	14.7
>£50 000 pa	45.9	30.8
Season measurement taken		
Summer	17.7	18.5
Autumn	49.7	47.3
Winter	20.5	21.7
Spring	12.2	12.5

### Active travel by distance-to-school and walkability (cross-tabulations)

The median home-to-school distance was 1.1 km (Inter Quartile Range: 0.64–1.73). Table 2 shows the proportion of children travelling actively to school for all journeys, and for 60% or more of school journeys, by home-to-school distance category and walkability quintile. Less than half of all children reported active all travel (n=304, 42.7%), and around two-thirds (n=468, 65.7%) active 60%*+* travel, to and from school (ie, both directions combined).

**Table 2:** Proportion of children actively travelling all (active all) and most (active 60%+) school journeys by distance category and walkability quintile (weighted)

No of children		Proportion active all journeys	Proportion active most journeys
(a) Home-to-school distance			
<0.5 km	126	78.6	86.3
0.5 to <1 km	236	49.5	79.6
1 to <1.5 km	150	30.7	57.9
1.5 to <2 km	73	26.0	49.6
2 km or more	128	18.0	37.8
Total	*713*	*42.7*	*65.7*
		X² (p)=120.2 (p<0.001)	X² (p)=100.9 (p<0.001)
(b) Walkability			
Most walkable	133	57.8	84.5
2	125	41.7	68.5
3	120	40.0	59.6
4	151	37.8	63.0
Least walkable	184	38.0	56.5
Total	713	42.7	65.7
		X² (p)=15.9 (p=0.1035)	X² (p)=30.7 (p=0.0081)

With increasing distance, the proportions of children actively travelling to school for all journeys decreased; 78.6% of children who lived within 0.5 km network distance of their school travelled active all, reducing to 18% of those living 2 km or more from school. Around 86% of children living within 0.5 km travelled actively 60% or more school journeys, with the proportion reducing to over a third of those living more than 2 km from school.

In terms of walkability and active all, the greatest proportion of active travellers for all school journeys was in the most walkable areas (Q1: 57.8%), and the smallest proportions within the second least (Q4) and least walkable (Q5) areas (37.8% and 38.0% respectively). For active 60%*+*, the highest proportion was seen in the most walkable areas (84.5%) and the lowest proportion within the least walkable areas (56.5%).

### Logistic regression models

The likelihood of a journey to school was modelled for active all and active 60%+. The models were performed separately for home-to-school distance and walkability of the home neighbourhood, and a third model included both walkability score and distance. Table 3 presents the results of the adjusted models (unadjusted results are available within an online supplementary table as unadjusted results vary little from adjusted results).

10.1136/bmjopen-2019-033628.supp2 Supplementary data

**Table 3:** Likelihood of school journeys using active travel by home-to-school distance and walkability of home neighbourhood (weighted)

Home-to-school distance					Walkability				
	Active all		Active 60%+			Active all		Active 60%+	
	OR (95% CI)	P value	OR (95% CI)	P value		OR (95% CI)	P value	OR (95% CI)	P value
Adjusted for urban/rural, income, season and latitude									
<0.5 km	Ref				1 (most walkable)	Ref			
0.5 to <1 km	0.22 (0.10 to 0.48)	<0.001	0.58 (0.20 to 1.67)	0.304	2	0.73 (0.32 to 1.67)	0.452	0.44 (0.21 to 0.91)	0.028
1 to <1.5 km	0.09 (0.04 to 0.20)	<0.001	0.19 (0.07 to 0.51)	0.001	3	0.52 (0.22 to 1.23)	0.136	0.29 (0.12 to 0.70)	0.007
1.5 to <2 km	0.09 (0.04 to 0.22)	<0.001	0.14 (0.05 to 0.37)	<0.001	4	0.51 (0.25 to 1.06)	0.071	0.33 (0.15 to 0.70)	0.005
2 km+	0.05 (0.02 to 0.11)	<0.001	0.09 (0.03 to 0.21)	<0.001	5 (least walkable)	0.45 (0.21 to 0.99)	0.047	0.20 (0.07 to 0.47)	<0.001
Adjusted for urban/rural, income, season and latitude; walkability/distance within same model									
<0.5 km	Ref.				1 (most walkable)	Ref.			
0.5 to <1 km	0.23 (0.11 to 0.51)	<0.001	0.67 (0.24 to 1.82)	0.422	2	0.70 (0.30 to 1.67)	0.421	0.37 (0.17 to 0.80)	0.012
1 to <1.5 km	0.10 (0.04 to 0.21)	<0.001	0.21 (0.08 to 0.53)	0.001	3	0.59 (0.25 to 1.39)	0.222	0.31 (0.14 to 0.70)	0.005
1.5 to <2 km	0.10 (0.04 to 0.22)	<0.001	0.15 (0.06 to 0.38)	<0.001	4	0.65 (0.32 to 1.30)	0.218	0.36 (0.16 to 0.81)	0.014
2 km+	0.05 (0.03 to 0.15)	<0.001	0.11 (0.05 to 0.25)	<0.001	5 (least walkable)	0.68 (0.31 to 1.52)	0.341	0.26 (0.11 to 0.64)	0.004

### Home-to-school distance

The likelihood of travelling actively on all school journeys (active all) decreased with increasing distance. Compared with the reference category (distance <0.5 km), children living 0.5 to <1 km, and those living 1 to <1.5 km from school, were around 80% and 90% (respectively) less likely to be involved in ATS for all their journeys (active all) (0.5 to <1 km: OR 0.22 (95% CI 0.10 to 0.48); 1 to <1.5 km: OR 0.09 (95% CI 0.04 to 0.20)). For the longest distances, further reductions in likelihood were seen (1.5 to <2 km: OR 0.09 (95% CI 0.04 to 0.22); 2 km or more: OR 0.05 (95% CI 0.02 to 0.11)).

Compared with those living within 0.5 km of school, those living further away (except 0.5 to <1 km) were less likely to use ATS for most journeys (active 60%*+*) (1 to <1.5 km: OR 0.19 (95% CI 0.07 to 0.51); 1.5 to <2 km: OR 0.14 (95% CI 0.05 to 0.37); 2 km or more: OR 0.09 (95% CI 0.03 to 0.21).

### Walkability

The odds of active all were significantly less likely for those living within the least walkable areas only (OR 0.45, 95% CI 0.21 to 0.99). Those within the least walkable neighbourhoods showed the lowest odds of active 60%*+* (OR 0.20, 95% CI 0.07 to 0.47), followed by those living within Q3 (OR 0.29, 95% CI 0.12 to 0.70), Q4 (OR 0.33, 95% CI 0.15 to 0.70) and Q2 (OR 0.44, 95% CI 0.21 to 0.91) of walkability.

### Home-to-school distance and walkability combined

In comparison to those living closest to school (<0.5 km), those living >0.5 km were less likely to active all, and those living 1 km or more were less likely to active 60%*+*. There were no significant associations between walkability and active all. For active 60%*+* those within the least walkable areas showed the lowest odds (OR 0.26, 95% CI 0.11 to 0.64), followed by Q3 (OR 0.31, 95% CI 0.14 to 0.70), Q4 (OR 0.36, 95% CI 0.16 to 0.81) and Q2 (OR 0.37, 95% CI 0.17 to 0.80).

### Interaction plots

**Figure 1:**
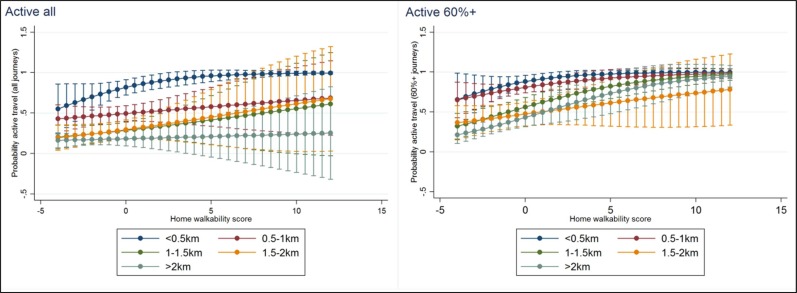
Predicted probability of active travel by walkability score and proportion of journeys active. (Note that some of the predicted probabilities have CIs that exceed 0/1: these should be taken as bounded at these limits. Walkability scores are Z-Scores. So contain both negative and positive values.)

Figure 1 displays interaction plots of the marginal effects, illustrating the predicted probability of active all and active 60%*+* by each distance category, at different values, along the range of walkability density scores. In general, the odds of ATS are higher where the distance travelled is shorter, as expected. However, it is interesting that (1) increasing walkability within an area increases the probability of ATS within all distance categories; and (2) this is not a constant increasing linear effect, with reduced gains after a certain point. For the likelihood of active 60%*+*, walkability eliminated differences between the longer distances and the shortest.

## Discussion

In our study, we explored whether home-to-school distance and home neighbourhood walkability score were associated with self-reported ATS among a sample of children aged 10–11 years old living within Scotland. We found that the likelihood of using an active mode of travel for *all* or *most* school journeys generally decreased with increasing home-to-school distance, and ATS was less likely for those living within the least walkable areas. We also found that the walkability score of the home neighbourhood had a larger effect on the probability of most school journeys as active, than the probability of always travelling in an active manner. The results are particularly notable in that the relationship between walkability and ATS persisted irrespective of home-to-school distance (and urban or rural location, household income, season and latitude (as a proxy for weather)).

### Home-to-school distance

Our findings correspond with findings of previous studies: those with a shorter travel distance to school were more likely to travel actively.^20 24 27 32 34 36 37 39–45^ Similarly to our findings, a study of children in a region of Denmark showed that those living within a half kilometre of school were most likely to use ATS,^42^ while other research based in Australia and Spain found a slightly higher threshold of around three-quarters of a kilometre.^27 30 45^ In our research, we found that although the majority of children living nearby (ie, within 0.5 km or roughly a 6 min walk) travelled actively, around 20% did not use ATS for all journeys and 15% did not do so, even for most school journeys. Although the influence of distance on ATS is unequivocal, additional factors are in operation which influence travel mode choice.

When the interaction between walkability and distance were included in the model we found that, for all distance categories, higher walkability increased the probability of ATS (for most school journeys). Similarly, Christiansen *et al* found this to be the case for all distance categories, with the exception of <0.5 km, where ATS rates did not differ between low or medium-to-high walkability areas.^42^ Ikeda *et al* found evidence of a dwelling density and home-to-school distance interaction.^31^ Low dwelling density and low distance combined were positively associated with ATS in their study. However, the authors asserted that a short distance to school may take precedence over dwelling density within this association, and further investigation of other relevant factors, such as traffic and road safety, was needed.^31^ Our findings suggest that the relationship between walkability score and ATS is more complex than the clear-cut association between home-to-school distance and ATS. A more walkable environment may be less important to those who walk or cycle every trip to school, and improving walkability may be of greater benefit to those who actively travel most of the time.

### Walkability

In accordance with our work, numerous pieces of existing research reported positive relationships between residential density and intersection density and ATS rates, whether analysed as individual components or within composite walkability scores.^22 27 37 38 42 46^ In contrast, selected Canadian research found residential and intersection densities to be negatively related to ATS^23 24^; the authors speculated that these factors were correlated with increased traffic and a greater number of roads for children to cross en route, which deterred walking. From our analysis, we cannot determine why the density factors were associated positively with ATS, but higher residential density could suggest a higher number of structural supports for walking, such as pavements and street crossings.^20^ Greater numbers of residences in a neighbourhood could also amplify feelings of a safe ‘social environment’ with more people in view on the street.^40^ Indeed, previous research argued that observing other walkers and cyclists on neighbourhood streets increased the likelihood of actively commuting to school among Brazilian adolescents.^63^ Furthermore, higher intersection density could facilitate ATS by allowing shorter and more direct routes with less need for detour,^44^ fewer dead ends and potentially a greater choice of routes. A choice of various alternative routes for getting from place to place in a neighbourhood was perceived as a beneficial factor for active commuting to school in a study of children and their parents in Melbourne, Australia.^64^

A Spanish study^38^ and a Canadian study^22^ noted higher rates of ATS in highly walkable areas, in comparison to less walkable areas, however, the latter study stated that this was true for high income areas only. Parents within low-income/high-walkability areas had greater worries about children’s safety, and the authors proposed this may lead to lower rates of ATS in these particular areas. Adjusting for income within our models did not alter significance levels, however, we did find that a greater proportion of children from lower income households used ATS for all journeys (61%), compared with those from higher income households (37%). Previous Scotland-based research also found that children in low-income areas were the most likely to walk or cycle to school^65^; this was regardless of more deprived areas having the highest child road, pedestrian and cyclist casualties.^58^ From our analysis, we cannot ascertain why greater numbers of children from poorer households use ATS, but our previous research did find poorer neighbourhoods in Scotland to be more walkable.^47^

Carver *et al* established that although those living in areas of high walkability were more likely to walk than those in low walkability areas, there were additional factors beyond the built environment which influenced ATS. These factors included children being accompanied by an adult, and ‘trip chaining’, for example, school drop off becoming a part of an adult’s journey to work.^27^ Another study recognised the importance of built environment for ATS and argued that social support from friends and siblings for walking was a relevant factor in mode of travel for Spanish adolescents.^37^ Furthermore, children’s ‘independent mobility’, that is, unaccompanied travel in the local area, has been associated with ATS.^66 67^ Page *et al* reported that children aged 10–11 years old with greater levels of independent mobility showed higher participation within various physical activity contexts including ATS.^67^ Inclusion of data on adult accompaniment, trip chaining, peer support and independent mobility was beyond the scope of our study but may provide explanation as to why some children living very close to the school did not use ATS more regularly.

Existing research highlighted additional objective measures, which may be associated with ATS, such as traffic safety (eg, presence of major roads) or pedestrian safety (eg, availability of pavements/sidewalks). Findings on the relevance of these factors for ATS are mixed. In Finland, children were found to be less likely to cycle to school when there were higher numbers of major roads on the school route,^39^ while a Canadian study found that with increases in traffic and pedestrian safety (ie, greater lengths of sidewalks, lower traffic volume and more traffic calming measures) children’s daily minutes of ATS increased.^26^ A Californian study reported that children were more likely to walk to school where sidewalks were present,^18^ while other USA and Canadian-based research showed no such relationship.^19 23 25^ Presence of lower traffic roads around home and school was not associated with ATS in research undertaken in Melbourne and Australia,^27^ while other research in Southern Denmark, Canada and the USA found negative associations between traffic density and rates of ATS.^20 23 42^ Giles-Corti *et al* maintained that the benefits of well-connected streets would be reduced if such walkable areas also had high volumes of traffic: children were less likely to travel actively in areas which had both high connectivity and high traffic volume.^29^ New Zealand-based research noted the importance of ‘perceptions of safety’ in parental decisions about travel mode choice for their children; parental concern about traffic safety was negatively associated with ATS.^68^ Ikeda *et al* asserted that combining improved walking/cycling infrastructure with educational programmes to enhance children’s motor and cognitive skills, for safer active travel, could alleviate parents’ safety concerns. Future analysis could incorporate measures of traffic and pedestrian safety within a composite walkability score.

Our study displayed a number of strengths. Our analysis used a sample of children from across the whole of Scotland, and was weighted to ensure representativeness of the wider population of 10–11 years old in Scotland. We included both roads and paths in the intersection density measure within our walkability score, thus provided more realistic models of pedestrian movement; various other studies included road networks only. We included interactions between walkability score and home-to-school distance within models which allowed us to compare how walkability was associated with ATS among those who lived nearer and those who lived further from their school; many studies do not incorporate this type of comprehensive analysis. Regarding limitations, this study was cross-sectional, hence we cannot assume that associations between key variables are causal, and cannot assume that the findings of this study are generalisable within other contexts. Travel mode was determined via travel diary entries which may not be as accurate as objectively measured mode of transport. Our study may be limited by the inclusion of shortest home-to-school distance rather than actual distance which children travelled. In the future work, we plan to use the children’s accelerometry and GPS data to determine precise home-to-school distance, travel mode and travel time. We also recognise that use of data zone level walkability score may lead to bias from the ‘modifiable area unit problem’, that is, the potential for statistical bias from using arbitrarily classified units to report spatial patterning.^69^

## Conclusion

The findings of our nationwide study show that neighbourhood walkability scores are related to ATS, calculated for all or most school journeys, within all distance categories. The relationship between walkability score and ATS is more complex than the clear-cut association between home-to-school distance and ATS: a more walkable environment appears to have a larger effect on the probability of reaching the 60% threshold of school journeys using ATS than the probability of always travelling in an active manner. Investment is needed in existing less walkable neighbourhoods to provide the infrastructure to support opportunities for ATS. Those involved in developing urban and transport policies should work towards improved street connectivity. Education authorities should collaborate with planning and public health professionals, and consider dwelling density and school catchment size when siting schools.
