# Supplementary material for: Active commute to school: does distance from school or walkability of the home neighbourhood matter? A national cross-sectional study of children aged 10–11 years, Scotland, UK
Source: BMJ Open. 2019 Dec 23;9(12):e033628. doi: 10.1136/bmjopen-2019-033628 (PMC7008418; doi:10.1136/bmjopen-2019-033628)
Supplement: Supplementary data [file bmjopen-2019-033628supp001.pdf]

Supplementary text: Clustered and stratified survey sample design, and Sample weighting

Clustered and stratified survey sample design

GUS sample: A named sample of approximately 10,700 children was selected from the Child Benefit records to give an achieved sample of 8,000 overall. An area-level sampling frame was created by aggregating Data Zones to give an average of 57 births per area per year. The list of aggregated data zones was sorted by Local Authority and then by the Scottish Index of Multiple Deprivation Score. 130 areas were then selected at random. The Department of Work and Pensions sampled the children from these 130 sample points. Within each sample point, Child Benefit records were used to identify all babies and three-fifths of toddlers who met the required date of birth criteria. Sampling of children was carried out on a month-by-month basis in order to ensure that the sample was as complete and accurate as possible at time of interview. In cases where there was more than one eligible child in the selected household, one child was selected at random. Twins had an equal chance of being selected.

Sample weighting

Growing up in Scotland (GUS) SPACES was a follow-up study for Phase 1 participants of GUS Sweep 8 hence there were three types of respondents, namely: participants in GUS SPACES, non-participants who were given a chance to take part and non-participants who weren't given a chance to participate (GUS Phase 2 sample members). To account for:

i) the fact that GUS SPACES is only based on phase 1 and full productives;
ii) (non-) consent to take part in follow-ups;
iii) (non-) response to GUS SPACES.

Non-participants were treated as one group of GUS SPACES unproductives and one step calibration was made to adjust the achieved sample to totals. GUS sweep 8 longitudinal sample weights (w8_baby) were used as entry weights for calibration and development of longitudinal weights for GUS SPACES and the GUS sweep 8 cross-sectional weight.

As an outcome, two weights were developed for GUS SPACES.

1. **MRC_child:** A longitudinal weight for analysis of GUS SPACES data for children whose prime carer has responded at every earlier sweep of GUS
2. **MRC_fullC**: A cross-sectional weight that should be used for any cross-sectional (sweep 8/age 10) analysis of the GUS SPACES data (i.e. data collected about the child). All children that completed follow-up have a cross-sectional child weight.

For the purposes of describing the weighting, respondents in GUS SPACES have been named Sample A and Sample B. These samples are defined as follows:

- Sample A – children whose carers had responded at all previous sweeps 1-8
- Sample B – children whose carers had participated in GUS SPACES but had missed one or more interviews in GUS Sweeps 2-7.

The two samples were treated separately during weighting. This is because the Sample B (n=775) respondents are likely to have different characteristics to those in Sample A (n=737), as suggested by their much lower response rates.

Longitudinal weights (MRC_child)

Longitudinal weights were only generated for respondents in Sample A. Calibration weighting methods were applied which take the pre-calibrated weighted combined sample and adjusts the weights using an iterative procedure. The resulting weighting factors, when applied to the combined data, will make the survey estimates match a set of population estimates for a set of key variables. The population estimates in this instance are survey estimates from Sample A, weighted by the main GUS sweep 8 longitudinal weight (w8_baby). Since the longitudinal weight corrects for sampling error and non-response bias at each stage of GUS, the weighted Sample A estimates are the best population estimates available. The choice of the variables to use in the calibration was dictated by the bias remaining in the data after the SW8 longitudinal weights were applied to Sample A. Variables used in the calibration of the longitudinal sample were:- Respondent age, Last known tenure, Family type, Location of household, Scottish Index of Multiple Deprivation 15% most deprived data zones.

Cross-sectional weights (MRC_fullC)

Cross-sectional weights were generated for all GUS SPACES respondents (Sample A+B). Calibration weighting methods were applied which takes the pre-calibrated weighted combined sample and adjusts the weights using an iterative procedure. The resulting weighting factors, when applied to the combined data, will make the survey estimates match a set of population estimates for a set of key variables. The population estimates in this instance are survey estimates from Sample A+B, weighted by the GUS sweep 8 cross-sectional weight (w8_fullB). The choice of the variables to use in the calibration was dictated by the bias remaining in the data after the SW8 cross-sectional weights were applied to Sample A+B. As the difference between sample A and A+B is only of 38 observations the key variables used in the weighting are exactly same as for longitudinal solution.
