# Supplementary material for: Active commute to school: does distance from school or walkability of the home neighbourhood matter? A national cross-sectional study of children aged 10–11 years, Scotland, UK
Source: BMJ Open. 2019 Dec 23;9(12):e033628. doi: 10.1136/bmjopen-2019-033628 (PMC7008418; doi:10.1136/bmjopen-2019-033628)
Supplement: Supplementary data [file bmjopen-2019-033628supp002.pdf]

Supplementary table

Home-to-school distance					Walkability				
	Active all		Active 60%+			Active all		Active 60%+	
	OR (95% CI)	p-value	OR (95% CI)	p-value		OR (95% CI)	p-value	OR (95% CI)	p-value
**Unadjusted**									
**<0.5km**	Ref.				**1 (Most walkable)**	Ref.			
**0.5 to <1km**	*0.27 (0.13-0.54)*	*<0.001*	0.62 (0.23-1.68)	0.340	**2**	0.52 (0.23-1.18)	0.116	*0.40 (0.19-0.84)*	*0.016*
**1 to <1.5km**	*0.12(0.06-0.25)*	*<0.001*	*0.22 (0.09-0.54)*	*0.001*	**3**	0.49 (0.22-1.06)	0.069	*0.27 (0.12-0.59)*	*0.001*
**1.5 to <2km**	*0.10 (0.04-0.21)*	*<0.001*	*0.16 (0.06-0.38)*	*<0.001*	**4**	*0.44 (0.22-0.88)*	*0.021*	*0.31 (0.14-0.69)*	*0.005*
**2km+**	*0.06 (0.03-0.13)*	*<0.001*	*0.10 (0.04-0.22)*	*<0.001*	**5 (Least walkable)**	*0.45 (0.24-0.82)*	*0.011*	*0.24 (0.12-0.47)*	*<0.001*

OR=Odds Ratio, CI= Confidence Intervals, p-value = Calculated Probability (level of marginal significance).
